# Supplementary material for: Person–environment fit and medical professionals’ job satisfaction, turnover intention, and professional efficacy: A cross-sectional study in Shanghai
Source: PLoS One. 2021 Apr 27;16(4):e0250693. doi: 10.1371/journal.pone.0250693 (PMC8078800; doi:10.1371/journal.pone.0250693)
Supplement: S1 Questionnaire — (DOCX) [file pone.0250693.s003.docx]

**The Sixth National Health Service Survey**

**Medical Staff Questionnaire (Partial)**

1. Sex：□Male □Female
2. Age（years）：_____________
3. Marital status：□Not married（Including unmarried, divorced and widowed） □Married
4. Education：□PhD □Master's degree □Bachelor's degree □College □Vocational Technical School □Technical School □High school □Junior high school and below
5. Professional qualifications：□Physicians □Assistant physicians □TCM physicians □Assistant TCM physicians □Nurses □Others
6. Type of organization：□Hospitals □Community health service centers

| Please circle the number that best matches your status  0. = completely disagree 6= fully agree | | | | | | | | |
| --- | --- | --- | --- | --- | --- | --- | --- | --- |
| 7. | My personality is a good match for this job. | 0 | 1 | 2 | 3 | 4 | 5 | 6 |
| 8. | My job helps make me the person I want to be. | 0 | 1 | 2 | 3 | 4 | 5 | 6 |
| 9. | Work inspired me. | 0 | 1 | 2 | 3 | 4 | 5 | 6 |
| 10. | My motivation for work stems from loving this job. | 0 | 1 | 2 | 3 | 4 | 5 | 6 |
| 11. | I am the right type of person for this type of work. | 0 | 1 | 2 | 3 | 4 | 5 | 6 |
| 12. | I am passionate about this job. | 0 | 1 | 2 | 3 | 4 | 5 | 6 |
| 13. | I want to go to work when I go to bed in the morning. | 0 | 1 | 2 | 3 | 4 | 5 | 6 |
| 14. | My abilities fit the demands of this job. | 0 | 1 | 2 | 3 | 4 | 5 | 6 |

| Please circle the number that best matches your status  1. = completely disagree 4 = fully agree | | | | | |
| --- | --- | --- | --- | --- | --- |
| 15. | Employees can work in unity. | 1 | 2 | 3 | 4 |
| 16. | When making key decisions, my team members will consult me. | 1 | 2 | 3 | 4 |
| 17. | My skills and abilities match the skills and abilities this team looks for in team members. | 1 | 2 | 3 | 4 |

| Please circle the number that best matches your status  1. = completely disagree 6 = fully agree | | | | | | | |
| --- | --- | --- | --- | --- | --- | --- | --- |
| 18. | I am satisfied with the promotion and career development prospects in this job. | 1 | 2 | 3 | 4 | 5 | 6 |
| 19. | Considering my skill and efforts, I am satisfied with the level of my income. | 1 | 2 | 3 | 4 | 5 | 6 |
| 20. | I am satisfied with the welfare received at work. | 1 | 2 | 3 | 4 | 5 | 6 |
| 21. | I am satisfied with the training opportunities I got at work | 1 | 2 | 3 | 4 | 5 | 6 |
| 22. | I am satisfied with working conditions and equipment configuration. | 1 | 2 | 3 | 4 | 5 | 6 |

| Please circle the number that best matches your status  1. = completely disagree 4 = fully agree | | | | | |
| --- | --- | --- | --- | --- | --- |
| 23. | My work will have a greater impact on the lives or happiness of others. | 1 | 2 | 3 | 4 |
| 24. | The quality of my work will affect many people. | 1 | 2 | 3 | 4 |
| 25. | I feel that work has lots of requirements for my ability. | 1 | 2 | 3 | 4 |
| 26. | I have to take on a lot of responsibilities in my work. | 1 | 2 | 3 | 4 |

| Please circle the number that best matches your status  1. = completely disagree 6 = fully agree | | | | | | | |
| --- | --- | --- | --- | --- | --- | --- | --- |
| 27. | I want to leave this hospital. | 1 | 2 | 3 | 4 | 5 | 6 |
| 28. | I often want to leave the industry I am working in currently. | 1 | 2 | 3 | 4 | 5 | 6 |
| 29. | Recently I often want to change jobs. | 1 | 2 | 3 | 4 | 5 | 6 |
| 30. | I’m likely to find a new job next year. | 1 | 2 | 3 | 4 | 5 | 6 |
