# Supplementary material for: Person–environment fit and medical professionals’ job satisfaction, turnover intention, and professional efficacy: A cross-sectional study in Shanghai
Source: PLoS One. 2021 Apr 27;16(4):e0250693. doi: 10.1371/journal.pone.0250693 (PMC8078800; doi:10.1371/journal.pone.0250693)
Supplement: Attachment — Submitted filename: Response to Reviewers.docx [file pone.0250693.s004.docx]

**Dear editor Geilson Lima Santana,**

We are glad to receive your letter for the revision request. Manuscript ID PONE-D-20-01262 entitled "How does person–environment fit affect Shanghai medical workers’ job satisfaction, turnover intention, and sense of accomplishment? An exploratory cross-sectional study" which we submitted to PLOS ONE, has been revised. Thank you for your hard work and the comments from two reviewers. According to your request and reviewers’ advice, we have made a careful modification of our manuscript. The following is the list and explanation of the revision.

- The submitted manuscript has been modified according to PLOS ONE's style requirements.
- According to your request, we have attached the questionnaire in the supplementary information. However, it should be noted that this is the Shanghai part of the sixth national health service survey and the questionnaire are not allowed to be disclosed. For the needs of article review, we just provide the questionnaire to the editors and reviewers. Please keep it only for this manuscript, thanks.
- We have modified the ethics section in the online submission.
- According to your request, we have submitted all the data involved in this study in the supplementary information.
- We have amended the ethics statements in the Methods section of the manuscript. (see the Ethics approval and consent to participate part in red）

**Response to the reviewer 1：**

Thank you very much for your advice. According to your advice, we have made a careful modification of our manuscript. And all the revised parts are marked in red in our paper. The following is the explanation of the revision.

1. **The study explored the attitudes of Shanghai medical workers based on the PE fit theory and explore the intrinsic relationships and influential factors between attitudes, turnover intention, and sense of accomplishment. This study is really intersting, but what is your innovations and new discoveries? Or is it just to prove that this model is correct? Maybe it needs more clear to express this part.**

We hope to explore the antecedents that affect the attitude variables of medical staff, and provide managers with implementation paths that can be improved and intervened. According to the research reviews, it is found that the theory of person-environment fit is often used to explore the impact on attitude variables. Based on the previous research on P-O fit, this study hopes to further understand the impact of P-J fit and P-G fit on attitude variables. There are few cross-sectional studies on applying PE fit theory to Chinese medical staff. The results of this study also have practical significance for strengthening the management of clinical medical staff. (See the Background and Hypothesis parts in red)

2. **So many terms makes readers difficult to understand the logic and correlation between different terms. It needs to reorganise to make it more clear. Such job satisfaction, the measurement of job satisfaction included the environments, so what is difference between other relvant terms of envirnments?**

Thank you for your suggestion. It is true that the logical relationship in the previous version of the manuscript is not clear enough. For this reason, we have rewritten the content of the Background and Hypothesis section, hoping that it will be easy for readers to understand. The question concerning the environment in the measurement of satisfaction is "How satisfied are you with the working environment?" The working environment here is a relatively general concept. In the theory of person-environment fit, the specific PJ fit and PG fit scales are used to evaluate the fit between person with their job, and person with their group. They are two important components in the theory of fit between person and environment. (See the Background and Hypothesis parts in red)

3. **Line 107-110 "However, it is worth noting that if people’s respect and self-worth needs are not met, it will lead to a decline in their sense of accomplishment. This will not cause dissatisfaction and there will be no sense of burnout". This sentence seems like the part of discussion. The logic of literature review is not clear and it needs to reorganise.**

Based on your suggestions, we sorted out the logical relationships and rewrote the content of this part. (See the Hypothesis parts in red)

4. **Line 123-124 Theories of PE interaction were very influential in management studies. Maybe it needs to delete.**

Thanks for your suggestions, we have removed.

5. **Line 123-136 This part is too tedious. It should be more concise.**

We have rewritten the content of this part. (See the Hypothesis parts in red)

6. **Line 161 randomly selected according to the proportion of 5 for each district. Please recheck the grammer and number.**

According to your advice, we have revised the sentence. (See the paragraph 2 of Design in red)

7. **Line 162 first-line medical workers, what is meaning of first-line medical workers, how to distinguish other types of medical workers?**

First-line medical workers or frontline medical workers refer to clinicians and nurses who are in close contact with patients in clinical work, and are different from those engaged in scientific research and managers in hospitals. This may be due to the problem of non-native language expressions. In order to facilitate understanding, we have modified into clinical medical workers.

8. **Line 161-165 How did you guarantee randomness?**

On the one hand, it is a large-scale survey at the national level, the randomness of sampling is completely guaranteed. On the other hand, we use Runs test to verify the randomness of the sample. In accordance with the principle of stratified sampling, we grouped administrative regions and types of institutions, and introduced age as the time variable. We established research hypotheses separately to verify the randomization of each group. It was found that the P value of each group was greater than 0.05.

9. **Line 165-166 What is the number of person who refused the survey?**

We hope to answer your question. However, it is a national-level investigation, and we are not authorized to disclose this information. Under normal circumstances, the investigation conducted by the National Health Commission will be highly cooperative.

10. **Table 1 The definition of Physicians in China is very different from that in this study. Please recheck the definition.**

The classification in Table 1 is more accurately described as qualification. In order to unify with the existing research, we merged it and modified the classification method involved in the subsequent analysis. (See the Table1 in red)

11. **Line 35-37 How did you make such findings? It did not appear in your tables.**

Through hierarchical regression analysis, we included the age variable in model, and according to the results we found age had a positive predictive effect on PJ fit and a negative predictive effect on PG fit. In order to fully explain the results of the study, we have supplemented the interpretation of the results. (See the last paragraph of Results in red)

12. **Line 41-43 " At the same time, new employees tend to be less adapted to the complex clinical environment and lack experience in dealing with physician–patient relationships and coordinating resources. " Can the SEM make such conclusions?**

According to the results of stratified regression, we found that younger physicians had lower PJ-fit. The above sentence is speculating on the possible reasons for this result, and the purpose is to provide a basis for subsequent intervention and improvement measures.

13. **The descriptions of PE fit is not clear, it should be desribed usuing more detail words, not professional terms.**

We try to clarify the concept of PE fit in the revised manuscript and the different dimensions it contains as much as possible. Hoping it can help readers better understand the meaning.

**Response to the reviewer 2:**

Thank you very much for your detailed instructions and suggestions. According to your advice, we have made a careful modification of our manuscript. And all the revised parts are marked in red in our paper. The following is the explanation of the revision.

1. **First, though the relevance of the problem they are addressing seems obvious, the authors are not being explicit about it, nor creating a clear link between the problem they want to address and the solution they aim to provide. In this sense, the authors claim they propose to bring empirical evidence by exploring “the attitudes of Shanghai medical workers based on the PE fit theory and explore the intrinsic relationships and influential factors between attitudes, such as job satisfaction, turnover intention, and sense of accomplishment” (page 5, second paragraph). However when justifying the relevance of the study they claim the need for theoretical development, which they do not provide in the manuscript (see the end of page 3: “it is of theoretical significance to further explore the antecedents of job satisfaction of medical workers”)**

According to your suggestion, in order to further clarify the problem to be solved and the internal relationship between variables, we have reorganized the background and the content of the research hypothesis, and supplemented the source and basis of the research hypothesis one by one. We hope that readers can better understand the purpose and content of the research.

First of all, we put forward the concept of PE fit and its prediction of the variable of working attitude. Then we clarify the focus of this study on PJ fit and PG fit; we explain the concept and development of PJ fit and PG fit respectively. In the research hypothesis section, we explained the basis and development of each hypothesis and the specific content in our research. (See the Background and Hypothesis parts in red)

2. **Second, the authors should provide a deeper and more accurate revision of the theoretical background of their study. Specifically they need to review the concepts of Personal Accomplishment, turnover and Person-Environment fit. Regarding Personal Accomplishment, I suggest the authors review in depth the theory of burnout proposed by Maslach, which they use to justify the construct of personal accomplishment. In this sense, the authors writing leads to misinterpretations on the relation between different constructs. As an example of this, in page 10 third paragraph the authors report: “Professional accomplishment and burnout are opposite definitions, but they have some relationship”. However, according to extant literature on burnout, personal accomplishment is one of burnout dimensions, because of this it is clear they are related. Thus, I suggest authors review again Maslach et al 2001 and Maslach and Leiter, 2008, which are part of the references. The review the authors make on these components must include also an examination on the instrument they are using to assess personal accomplishment, which comes from the Maslach Burnout Inventory-General Survey. As the authors should report in their manuscript, this version of the MBI does not assess Personal Accomplishment, but a different construct named “Professional Efficacy” which is less related to a sense of personal fulfilment and more related to a job related sense of self-efficacy. In this sense, their results are not related to accomplishment as they claim, but to professional efficacy. A second example of some of the confusions the writing of the manuscript leads to is that, to my knowledge, no literature propose any relation between Maslow’s theory of motivation and burnout dimension of professional efficacy (or personal accomplishment). If the authors do know any literature that propose these links I urge them to make proper cites and use the references, otherwise they should further develop their reasoning to propose such links. Regarding the literature on turnover and Person-Environment fit, the authors should devote a portion of the introduction and the theoretical background to define the constructs and include relevant empirical research that is pertinent to the development of their hypotheses.**

Probably due to non-native writing, the expression in the previous manuscript may indeed be problematic. We apologize if it is difficult for you or other readers to understand or cause misunderstanding. Thank you very much for your detailed and professional advice. In the revised manuscript, we have improved the concept of variables and the description of the relationship between variables, and at the same time supplemented the relevant research content of the research hypothesis. Regarding the part of job burnout, we must admit that this is indeed one of the limitations of this study. In our questionnaire, only one dimension of job burnout is involved (that is sense of accomplishment). Therefore, this study only discusses the impact of PE theory on a single dimension of job burnout. Since it is a national survey, we failed to add two other dimensions to the questionnaires. However, we hope to improve it in our future research. Regarding the relationship between Maslow’s theory of motivation and burnout as you mentioned, we have indeed found some related studies. ([1] Hale A J, Ricotta D N, Freed J, et al. Adapting Maslow's hierarchy of needs as a framework for resident wellness. Teaching and learning in medicine, 2019, 31(1): 109-118.[2] Gao Hong. Looking at nurses’ job burnout from Maslow’s hierarchy of needs theory[J]. Contemporary Medical Essays (Chinese), 2015, 13(16): 3-4.) Due to the re-writing of the background part, we have adjusted this content appropriately.

3. **Third, the authors will want to review the development of their hypotheses and provide further justification for each one of them. For example, it is not clear why in H1 the authors look for a relationship between Person-Group fit and the dependent variables of job satisfaction and accomplishment, whereas in H2 they look for a relationship between Person-Job fit and the outcomes of job satisfaction and turnover intention. Thus, it is not clear why H1 does not include turnover intention in their predictions and why H2 does not include accomplishment. Neither has it been clear why the authors proposed a mediation hypothesis in H3. The hypotheses should be driven from previous theory and empirical research that the authors articulate with their own aim. One way of achieving this is by addressing the second major revision, which was presented in the previous paragraph.**

Thank you for your valuable suggestions. We have rewritten and sorted out the content of the research hypothesis, hoping to explain as much as possible why we determined the above research hypothesis. (See the Hypothesis parts in red)

4. **Fourth, the pertinence of the analyses performed, and therefore the results presented is not clear. Also because of the previously named issues related to major problems in the hypotheses, it cannot be said that the results support the hypotheses (this is because it is not clear what the authors are hypothesizing about). Some recommendations to improve this aspect include: (i) The authors should provide specifications on the type of factor analyses performed. (ii) There is no presentation of dependent and independent variables descriptives (The descriptive statistics presented are the description of the sample, this should be moved to the participants subsection in the methods section). (iii) The authors should consider better analyses approaches, such as multivariate and regressions that include control variables such as age, gender and tenure (this is only presented in the hierarchical regression models, but the results are not clearly explained. (iv)Hierarchical regression models for PJ fit should also include education level, or specify why it does not consider it.**
(i) The results of the reliability and validity analysis of each dimension have been presented in the Instruments section.
(ii) We added a statistical description of the dependent and independent variables in the results section and moved the sample description to the methodology section. (see the paragraph 1 of Results and Participants of Methods in red)
(iii) The reason why it was not included as control variables is that after a large number of literature reviews, we did not find that these variables have an absolute impact on PG fit, PJ fit, job satisfaction, turnover intention, and sense of accomplishment. Nevertheless, we still use generalized sensitivity analysis to explore the impact of omit variables. According to each research hypothesis, we test the effect values of the omit variables separately. (Fig 1-3) Because the value of standardized coefficients of unobserved confounders in Hypothesis 1(b) and Hypothesis 2(a) are too small, it is unable to generate related graphs of GSA. The results of the sensitivity analysis show that the variables of age, gender, education, and occupation have very small impact. Finally, we incorporate these four variables into the structural equation model as control variables. The results of model fitting before and after adding control variables have been shown in Table 1. We found that the results after including the control variables were not better than before. We speculate that this may be related to the study design. Since the study adopted the principle of stratified random sampling and strictly adopted randomization in the sample selection process, the influence of covariates was not significant in this study. We simplifed the above process and add it to the methodology section. (See the Analysis in red)

Figure 1 GSA of Hypothesis I (a)

Figure 2 GSA of Hypothesis 2 (b)

Figure 3 GSA of Hypothesis 3

Table 1 The results of model fitting before and after adding control variables

|  | Before | After |
| --- | --- | --- |
| CMIN/DF | 6.438 | 8.063 |
| RMSEA | 0.046 | 0.053 |
| GFI | 0.950 | 0.929 |
| AGFI | 0.936 | 0.912 |
| TLI | 0.962 | 0.935 |
| CFI | 0.965 | 0.943 |

(iv) In the analysis process, we considered the three variables of age, occupations and education. But after adding the variable of education, the value of R^2^ had not been changed, and the significance of this variable was 0.179. Therefore, the final model does not include the variable of education. We have supplemented the above content in the results section, which will help the reader understand.
5. **Review the need to make an introduction explaining attitudes in psychology, at the moment it seems unnecessary.**

Due to the rewriting of the background section, this content has been deleted according to your suggestions.

6. **The authors should make explicit how prejudice was minimized (as proposed in page 7).**

The data for the analysis were drawn from the Sixth National Health Service Survey. The National Health Service Survey began in 1993 and is conducted every five years. The survey is organized by the National Health Commission, the Planning Department is responsible for coordination, and the National Statistical Information Center is responsible for the organization and implementation. They organize investigators to conduct unified training, investigation, data entry, quality control, supervision, etc. In order to fully grasp the situation in China, several rounds of expert consultations have been conducted to revise the survey plan to minimize prejudice and strictly implement the principle of randomized sampling. The investigators were systematically trained before the survey process. No hospital supervisor was involved in the whole process to ensure the authenticity of the questionnaire data.

7. **In page 7, paragraph 2 “physicians were randomly selected from the community health service center according to the number of workers”. It should be explained in greater depth how this selection was done and how many workers were part of these centers.**

The data for the analysis were drawn from the Sixth National Health Service Survey, which was provided by Shanghai Municipal Health Information Center. Because we were not involved in the sampling process, there is no more detailed information to provide, and also we were not informed about the number of employees in each center. However it is a large-scale survey at the national level, the randomness of sampling has been completely guaranteed.

8. **No explanation on how the participants were recruited is provided.**

The survey is organized by the National Health Commission. The Planning Department is responsible for coordination, and the National Statistical Information Center is responsible for the organization and implementation. They organize investigators to conduct unified training, investigation, data entry, quality control, supervision, etc. Shanghai Municipal Health Information Center provided us with the data in Shanghai.

9. **Example of the items that were develop for PJ and PG constructs should be provided.**

According to your request, we have attached the questionnaire in the supplementary information.

10. **Regarding the analyses performed major clarity and justification of the analyses performed should be presented (specially regarding the structural equation model).**

Through literature review, we found that regression analysis and structural equation models are usually more commonly used analytical methods to verify the relationship between PE fit and attitude variables and related research hypothesis.

11. **Descriptive statistics for the dependent and independent variables must the presented.**

According to your advice, we had added a statistical description of the dependent and independent variables in the results section. (see the paragraph 1 of Results in red)

**12.The discussion should not include results.**

According to your advice, we had removed the results from the discussion.

**13.The implications of the study should be further explored.**

The relevant content has been clarified and supplemented in the part of implications. (See the Implications part in red)

**14.The limitations of the study should include the limitations of self-reported questionnaires.**

According to your advice, we added the content above into the limitations. (See the Limitations part in red)

In spite of our best efforts, may the shortcoming is still existed, but we think it could be good reference for the researchers and policy makers. Thank you so much for your valuable advice again.

Yours sincerely,

Guohong Li

School of Public Health, Shanghai Jiao Tong University School of Medicine, Shanghai, China

Center for HTA, China Hospital Development Institute, Shanghai Jiao Tong University, Shanghai, China

guohongli@sjtu.edu.cn
