# Supplementary material for: Person–environment fit and medical professionals’ job satisfaction, turnover intention, and professional efficacy: A cross-sectional study in Shanghai
Source: PLoS One. 2021 Apr 27;16(4):e0250693. doi: 10.1371/journal.pone.0250693 (PMC8078800; doi:10.1371/journal.pone.0250693)
Supplement: Attachment — Submitted filename: Response to Reviewers.docx [file pone.0250693.s005.docx]

**Dear editor Geilson Lima Santana,**

Thank you for the opportunity to revise and resubmit the manuscript entitled "How does person–environment fit affect Shanghai medical workers’ job satisfaction, turnover intention, and sense of accomplishment? An exploratory cross-sectional study". We appreciate the insightful comments from the reviewer, and have made substantive revision base on the reviewer’ s advice.

**Response to the reviewer #2：**

Thank you very much for your detailed instructions and suggestions. Based on the comments, we have revised our manuscript. And all the revisions are marked in red in the paper. Here we provide our explanation point by point to address your comments.

1. **Even though you restructured the introduction and part of the hypothesis section, the changes you made do not respond to my first inquire: the problem you are solving and its relevance is still not explicit in the document. Lines 68 and 93 make me think this is a replica or further extension of a previous study, however the results from that study are not mentioned in the paper, and the relevance of replicating or extending it are not present in the document. In plain language, why is this an article that some other researcher would want to read? Why is this article valuable? And What are your contributions to the literature? This should be explicit in the introduction, and the hypothesis section should be devoted to explain how you derive each hypothesis from a theory and previous data (the theoretical component is still missing)**

Response: Thanks for the comments. We have revised the background section by adding research purposes and possible contributions to the literature. We have also summarized the results from previous research, and provided reasons for choosing PJ and PG fit as study variables in this study.

This is study is an extension of a previous study. This study is unique for two reasons: (1) most PE fit studies have been in the Western context and in the industrial setting, and we extend the PE fit theory to the health setting in the Eastern context; and (2) This study focuses on medical staff in Shanghai, and examines PE fit and job outcomes in a high workload setting. We have added following sentences in the introduction section: “*Most PE fit studies have been conducted in the Western context and in the industrial settings. In this study, we extend the PE fit theory to the health setting in China” and “Shanghai provides a good setting to examine factors, including PE fit, associated with job retention and satisfaction.”*

Additionally, we have added the following paragraph on the potential contribution of this study: “*To our knowledge, this is the first PJ fit and PG fit study on medical staff at the city level in China. It adds existing literature on the impact of the PE fit on job outcome variables among health professionals that require strong team-work and under high pressure. Given prevalent issues of a shortage and high turnover of health professionals in developing countries, this study provides empirical evidence on the impact of PE fit on job-related outcomes.*”

We also appreciate your comments on hypotheses. We have revised them and provided more detailed explanation of them. In each research hypothesis, we have included the relevant theories. for example, we have applied to hypothesis 1 the input-process-output model (IPO), which shows that the process (e.g., teamwork, coordination) can have a positive impact on the team output (e.g., job satisfaction, team performance, and profession efficacy), to hypothesis 2 the *Self-Verification Theor^y^ and Attraction Selection-Attrition model, and hypothesis 3* the conservation of resources (COR) theory. For detailed revision, please see our response to comment 12 and 13.

**2. Line 55, you need to first express what PE is (Person Environment fit?) before using an abbreviation (PE), this is valid also for line 59 PJ and PG.**

Response: Following your suggestion, we have spelt out “PE” before using it. The revised sentence reads as “*Person–environment (PE) fit is broadly defined as the compatibility between the individual and their work environment, which occurs when their characteristics match well.^2^*” We also spelt out PJ as person-job and PG as person-group.

**3. Lines 62-65 are not clear, what do you want to express?**

Response: We have further clarified the sentences to strengthening the argument that PE assessment has been applied throughout individuals’ career cycle. The revised sentences read as “*In the pre-hire stage, the measurement of PE fit was often used in career counseling, job searching and selection.^5^ At the same time, the majority of PE fit research has been conducted during the post hire period , showing strong linkage between single aspects of the PE fit and individual attitudes.^6,7^”*

**4. Lines 69-70 why did you choose those 2 dimensions, just because a different study selected other? You must have a better reason.**

Response: We have provided additional reasons why only PJ and PG were selected for the study. There are two major reasons:

1. PJ and PG fit are the concepts more concentrated on team work, which is a main characteristics of medical practice in China. These two concepts are more pertinent to the analysis among medical staff.
2. Compared to PO, PJ and PG fits are more modifiable in a relatively short period.

We have added a paragraph to explain why PJ and PG were selected for the study. The added paragraph reads as:

*“Most PE fit studies have been conducted in the Western context and in the industrial settings. In this study, we extend the PE fit theory to the health setting in China. Expanding the understanding of PE fit among medical staff is important, because most physicians in China are working in teams in health facilities. ^8,9^ Among the four components of PE, PJ fit and PG fit are closely related subordinate concepts that concerns the alignment of workers and their job as a team. In this study, we focused on PJ and PG fit, because (1) team-based care models are dominant in clinical practice in China, demanding for strong team cooperation.^10^ Studying PJ fit and PG fit can help understand how they impact medical staff’s job attitude and behavior; and (2) we had previously explored the impact of PO fit on job satisfaction and turnover intention among community health workers in China and found that PO fit had a little association with turnover intention.^11^ We posited that the values of individuals and organization were relatively difficult to change in a short period of time^12^. Focusing on PJ and PG fits allow us to examine factors more modifiable to address attitude concerns among medical professionals, which helps improve the quality of medical care.^13,14,15,16^”*

**5. Lines 75-77, why is this relevant? What did this author conclude?**

We aimed to provide the concepts of PJ fit and PG fit in the background section before getting into hypotheses. To make it clear for readers, we have added a subtitle of “the concept of PJ fit and PG fit”. We also revised the paragraph slightly to elaborate the three components in PJ fit. The revised paragraph now reads as “*PJ fit is defined as the consistency or match between the characteristics of a person and the work or tasks performed.^18^ Edwards suggested two fundamental components of the demands-abilities fit and needs-supplies fit falling within PJ fit.^1^ When the employee’s knowledge, skills and abilities (KSA) match the demand of work, the fit of demands-abilities occurs; when the work performed meets the employee's need, the fit of needs-supplies occurs.^19^ Later, Scroggins^20^ added the concept of "self-concept of work fit" as an additional component to the PJ fit. Self-concept of work fit means that when job tasks match the individual’s self-concept, the individual will perceive the work as meaningful.^20^*”

**6. Line 78, what do you mean by progress? The following paragraph is confusing**

We have revised the sentence to “*Compared with PJ-fit research, there are much fewer studies on PG fit.^22^*”. We also restructured and revised the whole paragraph.

**7. Line 83-85, why is the concept of team important? Support with previous research and literature.**

This paragraph means to introduce the concept of PG. To avoid confusion, we removed the sentence. However, we explained previously that the medical practice in China is mostly team-based, thus we wanted to examine PG fit in this study.

**8. Line 90, what do you mean by saying that the value between individuals and groups is similar (complementary)?**

We wanted to express that PG fit for the medical industry is mainly measured through value congruence, whereby the extent to which an individual shares similar values as their group members. To avoid confusion, we have removed the sentence.

**9. Line 95, this is a different idea, you need to break the paragraph.**

Following your advice, we have broken the paragraph.

**10. Lines 96- 103, you ignored my recommendation of not talking about personal accomplishment but professional efficacy, and elaborate an appropriate definition of the construct, however I didn’t find a satisfactory justification for this decision in your letter.**

Thank you for the suggestions. Following your suggestion, we have revised the whole paragraph and focused on the professional efficacy. The revised paragraph now reads as “*According to the input-process-output model (IPO), the process experience (e.g., teamwork, coordination) is associated with individual and team outputs (e.g., job satisfaction, team performance, and profession efficacy) ^24,25,26^: coordination and encouragement from team members enhance individual efficacy, which optimize the collective group effort.^26^ Previous studies also showed a relationship between team work and job satisfaction.^27,28, 29^ Group settings can change individuals’ motivation,^30,31^ and group variables (e.g. group composition, leader and group characteristics) might affect individual members’ sense of fulfilment towards their work.^32^ Medical practice in China is mostly team-based, requiring coordination of health providers within the same department or across different department*”

**11. Lines 103-106, seem completely disconnected from the previous ideas. This needs further elaboration to allow the hypothesis to become a conclusion of what you have been saying.**

We have revised the whole paragraph. See our response to comment 10.

**12. Lines 110-120 explain results from previous research but it doesn’t provide an explanation or derivation for the following hypothesis.**

Following your suggestion, we have focused on the explanation of the hypothesis and revised the entire paragraph.

The revised paragraph reads as “*Empirical studies have shown that PJ fit has an important impact on employees' work attitudes, such as job satisfaction and turnover intention.^33^ Smith^34^ found that job satisfaction was largely determined by employees’ work and their specific tasks. Caldwell’s study ^35^ suggested that satisfaction and performance were enhanced when individuals select a job that was compatible with his or her traits and skills. These findings were consistent with the self-verification theory^36^ and attraction selection-attrition model^37^ that predicted a negative relationship between meaningful work or the sense of job satisfaction and turnover intention. Meaningful work provides employees self-verified information, making the work itself attractive^38,39^. P-J fit has been established as an important predictor of job satisfaction and turnover intention, and we expect the same relationship will be held among medical staff in Shanghai.”*

*Hypothesis 2: Medical workers with higher PJ fit have: (a) higher job satisfaction and (b) lower turnover intention.*”

**13. Lines 133-135, I understand your aim is only to replicate the findings of Hassan. What is the reasoning for such relations to exist? And what are the implications or value of this study providing more evidence about it?**

Following your suggestion previously about the hypothesis, we have revised the entire paragraph by explaining the hypothesis using prior studies or theories. One of reasons for examining such relationship is to identify potential mitigation factors to address the concern of job turnover. We have added following sentences in the revised paragraph: “*Examining pathways on how PJ is associated with job turnover would help identify potential mitigation factors to address the concern of job turnover*.” And the revised paragraph on hypothesis 3 now reads as following:

*“Job satisfaction, reflecting the degree of personal enjoyment of work^40^, has shown its relevance to both individual job outcomes and organizational results, and it is a stable predictor of employee turnover intention and behavior.^41,42, 43,44^ Built on these findings and in combination with Hypothesis 2 that posits that PJ fit is associated with job satisfaction and job turnover directly, we further propose the third hypothesis of that job satisfaction could serves as a mediator between PJ fit and turnover intention, and PJ fit has both direct or indirect association with the turnover intention. In fact, Hassan M^45^ and Bindu Chhabra^46^ confirmed such relationship among employees in the Banking sector. Examining pathways on how PJ is associated with job turnover would help identify potential mitigation factors to address the concern of job turnover.”*

**14. Line 152-153, you should remove the statement about prejudice or make explicit that because of the characteristics mentioned above it was minimized.**

Thanks for your suggestions, and we have removed the statement.

**15. Lines 154- 167, now I really wonder if you were part of the survey collectors or if you received the collected information.**

We did not participate in the survey, but we obtained the collected information and learned how the survey was conducted. We have clarified it by stating that it is National Health Commission of China that conducted the survey, and that we obtained the relevant NHSS data from Shanghai Health Commission and used the survey data from Shanghai only for this study. We have revised the paragraph, which reads as “*We obtained the relevant NHSS data from Shanghai Health Commission and used the survey data from Shanghai only for this study. The survey includes all general hospitals and traditional Chinese medicine (TCM) general hospitals in 16 Shanghai districts. All tertiary hospitals were included in the survey; 50% secondary hospitals were randomly sampled and included in the survey; in each administrative district, 5 community health service centers were randomly selected. As to sampling health professionals, 10 clinicians and 5 nurses were randomly selected from each hospital, while 5 clinicians, 3 nurses, and 2 public health physicians were randomly selected from each community health service center. The survey contained a total of 2,600 health professionals. After eliminating missing values and logical errors from the samples, 2559 individuals were included in the analysis in this study.*”

**16. Line 172, what does it mean “considering workers in an organization”?**

We have revised it to “*In terms of the type of health facilities where health professionals worked*”

**17. Line 295 you make assumptions of causality which you cannot make with your design. Avoid words such as affect.**

Thanks for the suggestions, we have used association when discussing the results throughout the manuscript.

**18. Discussion line 13. Are you talking about you own results or results from different studies? You kept using words that imply causality such as effect.**

Line 13 is the results from a study from someone else. We have removed the statistics obtained from the study to avoid the confusion.

**19. Line 17 you talk again about effect**

We have corrected it, and it reads as “*We also found that PG fit was directly associated with professional efficac*y.”

**20. Line 19, why are you talking here about burnout if you did not develop these ideas in the introduction or background?**

We have removed burnout related sentence and revised the paragraph, which discusses more about the professional efficacy. The paragraph now reads as “*We also found that PG fit was directly associated with professional efficacy. A study found that there was a strong correlation between PO fit and professional efficacy in nonmedical fields.^52^ Few studies have explored the analysis of professional efficacy as a variable in the field of PE. With the introduction of the concept of team-based care, the in-depth study of PG fit is particularly urgent*. ”

**21. Lines 25-45, this is something new that you are contributing to the literature, you should spend more time developing this idea, and also introduce the relevance of those findings in the introduction… Also you should make explicit the analysis and results for this conclusion in the results sections even though there were not part of your hypothesis.**

Based on your suggestions, we have added one more objective in the background section. The added objective reads as “*Another purpose of this study is to explore individual characteristics associated with PJ and PG fit.*”

In the method section, we have singled out the hierarchical regression analysis by making it as a separate paragraph.

In the result section, we have added that a phrase of “*to explore factors associated with PJ and PG fit*” after the hierarchical regression analysis.

**22. Lines 35-38 find some literature that help you support these conclusions.**

Thank you for your advice. We have added three references in the manuscript. These references are:

53.Boamah S A, Laschinger H. The influence of areas of work-life fit and work‐life interference on burnout and turnover intentions among new graduate nurses. Journal of Nursing Management, 2016, 24(2): E164-E174.

54. Sun WB. Briefly on the difficulties and countermeasures in the training of young doctors in my country at this stage. China Hospital, 2011, 15(05): 66-69

55. Jiang SL. Cultivation and improvement of doctor-patient communication skills of young cardiovascular surgeons. China Journal of Modern Medicine, 2016, 18(01): 92-93.

**23. Lines 57-63 review the implications… is this realist? Is it better to suggest modifications in selection processes to find doctors who have a better fit to this job?**

Thanks for the suggestion. We have modified the sentence to “*The results of this study show that PJ fit is a critical factor determining job satisfaction and job turnover. It is important for hospital administrators to identify where the lack of fit lies between medical workers and work and to address it (e.g. through training), thereby reducing employee dissatisfaction and turnover intention. Research showed that turnover intention of medical staff was at a high level in China.^56^ Ensuring PE fit of health professionals might alleviate the persistent problems of labor shortage and frequent turnover of medical staff in China, which help improve the quality of medical care.*”

Additionally, we have also added “*In addition to the measurement of PJ fit when recruiting medical workers, it is also important to focus on strengthening the relevant fits among young physicians. It is possible to choose suitable position after rotation for individual employees by assessing the degree of fit between their job interests and the nature of their work. The medical professional association within hospitals or health facilities can also provide a communication platform for junior and senior physicians, which may be an effective way to improve their PJ fit as soon as possible.”*

**24. Lines 65-67, is not clear how you get to that conclusion.**

We have revised the sentence to “*Given prevalent issues of a shortage and high turnover of health professionals in developing countries and the strong association between PJ fit and job turnover intention, it is important to deepen research on PJ fit in order to better address these chronic problems*.”

We hope that we have thoroughly addressed the concerns that the reviewer has, and look forward to your decision.

Yours sincerely,

Guohong Li

School of Public Health, Shanghai Jiao Tong University School of Medicine, Shanghai, China

Center for HTA, China Hospital Development Institute, Shanghai Jiao Tong University, Shanghai, China

guohongli@sjtu.edu.cn
