# Supplementary material for: Person–environment fit and medical professionals’ job satisfaction, turnover intention, and professional efficacy: A cross-sectional study in Shanghai
Source: PLoS One. 2021 Apr 27;16(4):e0250693. doi: 10.1371/journal.pone.0250693 (PMC8078800; doi:10.1371/journal.pone.0250693)
Supplement: Attachment — Submitted filename: Response to Reviewers.docx [file pone.0250693.s006.docx]

**Dear editor Geilson Lima Santana,**

Thank you for the opportunity to revise and resubmit the manuscript entitled "Person–environment fit and medical workers' job satisfaction, turnover intention, and professional efficacy: An exploratory cross-sectional study in Shanghai". We appreciate the insightful comments from the reviewer, and have made substantive revision base on the reviewer’s comments.

**Response to the reviewer #2：**

Thank you very much for your detailed instructions and suggestions. Based on the comments, we have revised our manuscript. And all the revisions are marked in red in the paper. Here we provide our explanation point by point to address your comments.

1. **In the introduction you indeed mention that it is relevant to expand understanding on PE fit (lines 71-72) because most physician in the country are working in teams. However, you need to create closure of the idea you want to convey and be able to directly connect this fact (many doctors working in groups) with your theory. Also when you express the idea in that way you are presuming that in the western countries physicians work in isolation, and I am not sure that is the case (review the work of Gittell on Relational coordination).**

Response: Thanks for the comments. Following your suggestion, we deleted the sentence and elaborated on the theory of relational coordination. The revised sentence reads as “*Similar to the medical practice in Western countries, the medical practice in China often operates in teams and thus relational coordination among medical professionals directly impacts on the quality of care.*” We also cited Gittell’s work to strengthen the argument.

2. **You need to explain the relevance of your variable of interest before you propose the hypothesis, thus you need to explain before what those variables are and why it is important to find a way to improve them.**

Response: Thanks for the suggestion. We have restructured the introduction section substantially, and moved the definition of PJ and PG fit up to highlight these two variables as key independent variables, because they are more modifiable compared to PO fit. We also move the justification of using Shanghai as the study setting given the high workload among health professionals, and to highlight the three job outcome variables as main dependent variables.

3. **Review the limitations section, especially the last idea you present as it is incomplete.**

Response: We have revised the limitation section substantially. For example, the third limitation now reads as “*Third, we used self-reported questionnaires (tools) to measure key research variables in this study, and the reported results may be subjective to potential bias. For example, although we have tested the construct validity of questionnaires, participants can still provide untruthful responses, and it is not clear if the questioners used in this study have a good criteria validity to capture the essence of what we would like to measure. Future research should validate the questionnaires from other perspectives (e.g. criteria validity and content validity)*.”

4. **Make a thorough revision of writing, spelling and grammar, as some sentences are not clear (lines 27, 31, 38, 65, review line 287 when to talk about job achievement).**

Response: Thank you for your suggestion. We have made substantive editing: restructuring the introduction section; correcting grammar and spelling mistakes, and clarifying sentences.

We hope that we have thoroughly addressed the concerns that the reviewer has, and look forward to your decision.

Yours sincerely,

Guohong Li

School of Public Health, Shanghai Jiao Tong University School of Medicine, Shanghai, China

Center for HTA, China Hospital Development Institute, Shanghai Jiao Tong University, Shanghai, China

guohongli@sjtu.edu.cn
