# Supplementary material for: Person–environment fit and medical professionals’ job satisfaction, turnover intention, and professional efficacy: A cross-sectional study in Shanghai
Source: PLoS One. 2021 Apr 27;16(4):e0250693. doi: 10.1371/journal.pone.0250693 (PMC8078800; doi:10.1371/journal.pone.0250693)
Supplement: Attachment — Submitted filename: Response to Reviewers.docx [file pone.0250693.s007.docx]

**Dear editor Geilson Lima Santana,**

Thank you for the opportunity to revise and resubmit the manuscript entitled "Person–environment fit and medical workers' job satisfaction, turnover intention, and professional efficacy: An exploratory cross-sectional study in Shanghai". We appreciate the insightful comments from the reviewer, and have made substantive revision base on the reviewer’s comments.

**Response to the reviewer #2：**

Thank you very much for your detailed instructions and suggestions. Based on the comments, we have revised our manuscript. And all the revisions are marked in red in the paper. Here we provide our explanation point by point to address your comments.

1. **Regarding the major issue, please clarify the ethical implications analysis you made and report it in the corresponding section. Did the participants give their consent to collect the data and did they give permission to use it in research studies? What were the risks of participating in such study? Even if it does not represent a risk, you should make a statement about it. What does it mean that the study did “not require ethical approval”?... if the institutions that support this study does not require and IRB process, you should at least show a reflection about the ethical implications of this study for the participants, even if they are not patients or public, participants are human beings who must decide if they want to participate in studies or not. I kindly suggest that you review if the National Health Services Survey has made an analysis of the ethical implications of collecting the data and allowing to use it for research and if they collect informed consents from participants.**

Response: Thanks for the suggestion. What we want to express is that this study was based on the secondary analysis of existing National Health Services Survey datasets. The National Health Service Survey began in 1993 and is conducted every five years. It was organized by the National Health Commission, the Planning Department is responsible for coordination, and the Regional Health Information Center is responsible for implementation. All data in this study were obtained and available for research upon the approval from authorized Shanghai Municipal Health Information Center. The data in this study did not contain the identifiable private information.

The participants of original National Health Services Survey were adequately informed about all relevant aspects of the survey, including its objective and procedures. The survey had obtained their informed consent. All participants had the right to decide whether to participate in the survey, and could decide whether to withdraw at any time.

2. **101. “Later, Scroggins....” indicates that you are explaining the theory in a chronological way and you are not, simply avoid the later.**

Response: Following your suggestion, we deleted the conjunction.

3. **106-108. Two sentences explaining the same idea, please unify.**

Response: We have revised these sentences. The revised sentence reads as “*Compared with PJ-fit research, there is little research on antecedents of PG fit, and how it affects the performance of the team to which an individual belongs [13,14].*”

4. **109-110 The collaborative nature of medical practice is a good argument for studying PG fit, but not PJ fit. What is the argument for studying PJ fit?**

Response: Following your suggestion, we have added relevant content. The revised sentence reads as “*Given the collaborative nature of the medical practice and the professionalism and particularity of the work itself [15], we focused our analysis on the potential effect of PJ and PG fit on job outcomes, particularly on job satisfaction and employees’ intent to quit in Shanghai. Previous studies show that PJ and PG fit has a strong correlation with job satisfaction [1, 16].*”

5. **115-116 Review the way numbers are presented. Separate average number of patients from bed occupation.**

Response: Following your suggestion, we have separated average number of patients from bed occupation. The revised sentence reads as “*The reports showed that the average number of patients that a physician treats per day were 14.4 in Shanghai and hospital bed utilization were 95.85%.*”

6. **117-122 You present three different ideas without a context, or which are not connected to the rest of the paragraph: “Medical professionals have a high workload” and is a phrase out of context with the rest of the paragraph. “Maintaining a high level of job satisfaction has been challenging” is not connected with the rest of the ideas of the paragraph. “In this study we have not intended to assess the impact of PO fit” needs to be better connected with the following idea and makes part of a separate paragraph.**

Response: Thanks for the suggestion. We have restructured this paragraph and moved the sentence of “Maintaining a high level of job satisfaction has been challenging.” We also have separated the rest of the ideas into next paragraph.

7. **122-129 Please reformulate to make clear.**

Response: Thanks for the suggestion. We have reformulated this paragraph. The revised content reads as “*In this study, we have not intended to assess the impact of PO fit. We had previously explored the impact of PO fit on job satisfaction and turnover intention among community health workers in China and found little impact of PO fit on the turnover intention [19]. It was also found that the difference in the PO fit of the survey subjects was small, which may be related to that they were all from public medical institutions. [19] What’s more the values of individuals and organizations might be relatively difficult to change in a short time [20]. On the contrast, the PJ fit and PG fit are more modifiable to address attitude concerns among medical professionals, so as to improve the quality of medical care [21-24].*”

8. **176-180 It is not necessary to make a comparison with western countries, only to refer to the idea that working in coordination with others is central for the health care setting (regardless of the location). One way to do this is to use the concepts of relational coordination, but there might be other similar concepts or literature that refers to this notion.**

Response: Following your suggestion, we propose research hypotheses based on the concept of relationship coordination theory: *“Based on the theory of relationship coordination, it is also a form of organizing social capital, which can make it easier for people to obtain resources needed to accomplish one's work.[34] We know that having the resources needed to accomplish the work has been proved to be an important source of job satisfaction. [35] Medical practice is mostly team-based, requiring coordination of health providers within the same department or across different departments.”*

9. **190-191 If you refer to those theories you should briefly explain what they say in general, why are you using two theoretical models to explain the same thing? What are the differences between those theories?**

Response: Following your suggestion, we respectively introduced the specific content of these two theoretical models. The theory of self-verification showed that “*self-consistency improves the degree to which the individual feel that he can control and manipulate his surrounding environment. A stable self-concept allows individuals to negotiate social reality and understand how to act effectively in a given situation.*” And the attraction–selection–attrition model told us that “*individuals will be attracted to and seek out jobs and organizations that provide them with meaningful self-confirming information and will likely continue in the job as long as self-confirming information is received and a high level of self-concept–job fit is perceived.*”

10. **194-195 Please provide the cites and references for the studies that have established such relations.**

Response: Following your suggestion, we have added references as follows.

Tinsley HEA. The congruence myth: An analysis of the efficacy of the person–environment fit model. Journal of Vocational Behavior. 2000;56(2):147-79.

11. **234 What does it mean “ilogical errors from the sample”?**

Response: During the process of data cleaning, we found some logic errors. For example, there are two identical questions in the questionnaire to test whether the participants answered earnestly. If there was an inconsistency, the sample was regarded as a logical error.

12. **265 Separate the measures of PJ and PG. You use the subtitle PE fit, but you are measuring two of the three components of PE fit, and theorizing about them separately.**

Response: Following your suggestion, we have revised the subtitle into PJ fit and PG fit.

13. **267-279 How did you create these items? Are they adapted from a different survey or instrument? Please describe where does it come from and provide examples of the items.**

Response: Following your suggestion, we have added the process of revision and given a sample item of “*My personality is a good match for this job.*”

14. **280-284 Check grammar and spelling.**

Response: Thanks for the suggestion. We have revised this sentence. The revised content reads as “*The PG fit was measured using the scale constructed by Piasentin and Chapman (2007), and revised for medical workers. It included three aspects matching an individual and the team that the individual belonged to and was a 4-point Likert scale (1 = completely disagree; 4 = fully agree).*”

15. **291 –293 You refer to the components, but how many items did this scale included?**

Response: Thanks for the suggestion. This scale has 5 items. We have corrected in the manuscript.

16. **301 Be consistent in the writing... the previous paragraphs refer in passive voice to how the constructs were measured and in this paragraph and the next one you introduce the NHSS**

Response: Thanks for the suggestion. We have revised the sentence to keep consistent.

17. **308-323 You should only inlcude one sample item, by including all of them you are violating the terms and conditions of use of the MBI which should have been purchased.**

Response: Thanks for the suggestion. We have deleted the whole items and kept a sample item in each scale.

18. **316-317 An alpha of .84 is often considered good.**

Response: Thanks for the suggestion. The revised content reads as *“The Cronbach’s α was 0.840.”*

19. **329 You can eliminate the “calculate Cronbach coefficient to check internal consistency as this is what a Cronbach coefficient does.**

Response: Following your suggestion, we have removed these words.

20. **352-357 Please give some context on how to interpretate the values of the described variables... are they high, low, average compared to other samples using the same measures?**

Response: Thanks for the suggestion. Initially, we hope to compare these results between related studies in the discussion section. However, we found that some studies with the same measures have not presented these values, such as reference [1,2].

Some studies that presented these values showed that the degree of job satisfaction among teachers was 3.87 [3] and among employees was 3.61 [4]. The turnover intention of private sector employee was 3.16.[5] These studies were carried out in different countries and different industries. Due to the particularity of the sample, relatively few researches have been carried out in the medical field. This also increased the difficulty of comparison between the same measurement tools.

Our research had a reasonable sampling design, which made the results more representative. It can be said that this was the first PE fit study at the city level, and our results can reflect the overall situation of medical staff in Shanghai. We still retained this content in the results section, because we hope to let readers understand the distribution of our samples in these scales, and to provide a basis for follow-up research to compare with.

[1] Scroggins W A. Antecedents and outcomes of experienced meaningful work: A person-job fit perspective. The Journal of Business Inquiry, 2008, 7(1): 68-78.

[2] Wardana M C, Anindita R, Indrawati R. Work Life Balance, Turnover Intention, And Organizational Commitment in Nursing Employees at X Hospital, Tangerang, Indonesia. Journal of Multidisciplinary Academic, 2020, 4(4): 221-228.

[3] Ho C L, Au W T. Teaching satisfaction scale: Measuring job satisfaction of teachers. Educational and psychological Measurement, 2006, 66(1): 172-185.

[4] Moorman R H. The influence of cognitive and affective based job satisfaction measures on the relationship between satisfaction and organizational citizenship behavior. Human relations, 1993, 46(6): 759-776.

[5] Yin-Fah B C, Foon Y S, Chee-Leong L, et al. An exploratory study on turnover intention among private sector employees. International Journal of Business and Management, 2010, 5(8): 57.

21. **396. A mediating factor between what and what?**

Response: Thanks for the suggestion. The revised sentence reads as *“To validate Hypothesis 3, we constructed a pathway using job satisfaction as a mediating factor between PJ fit and turnover intention.”*

22. **398 Conclude the idea by specifying if the hypothesis was supported or rejected.**

Response: Thanks for the suggestion. The revised sentence reads as *“We found that PJ fit was indirectly associated with job turnover intention, and the association was statistically significant (**β=−0.185, P<0.001), which supported Hypothesis 3.”*

23. **2 Please start the discussion by posing again the main objective of the study. Also, in the discussion you should not use the specific results and statistics. Please remember that the discussion summarizes the main results by explaining its conceptual meaning. Because those statistics are already clear in the results section you should eliminate them from the discussion.**

Response: Following your advice, we have added the paragraph of the main objective of the study and deleted the specific results and statistics from the discussion. The revised paragraph reads as *“In keeping with the PE fit theory, we introduce a theoretical model that present hypotheses about the relations of PJ and PG fits with job satisfaction, turnover intention, and professional efficacy. Additionally, we explored the mediation mechanism of job satisfaction in PJ fit on turnover intention.”*

24. **In the discussion you should be clear about the theoretical or conceptual implications of your findings.**

Response: We have revised the whole paragraph. See our response to comment 23.

25. **35-36 You talk about improving PJ fit of younger physicians. How could that be done?**

Response: We have put forward practices and suggestions for reference in the implications section. The suggestion reads as “*In addition to the measurement of PJ fit when recruiting medical workers, it is also important to focus on strengthening the relevant fits among young physicians. It is possible to choose a suitable position after rotation for individual employees by assessing the degree of fit between their job interests and the nature of their work. The medical professional association within hospitals or health facilities can provide a communication platform for junior and senior physicians, where senior physicians can share their work experience and self-improvement methods with junior physicians through pairing or group sharing to improve their PJ fit.*”

26. **92-95 How would a communication platform help to improve PJ fit?**

Response: Thanks for the suggestion. We have added the explanation. The revised sentence reads as “*The medical professional association within hospitals or health facilities can provide a communication platform such as social media for junior and senior physicians, where senior physicians can share tacit knowledge according to the physicians’ perspectives and experiences [58]and self-improvement methods with junior physicians through pairing or group sharing to improve their PJ fit.*”

27. **115 As you did not conduct a study with an intervention, your study does not lead to the conclusion that strengthening PG fit could improve professional efficacy. Maybe your study allows to think that, but is not a factual conclusion. Please reformulate.**

Response: Thanks for the suggestion. The revised sentence reads as “*The results show that medical workers with higher PJ or PG fits have higher job satisfaction and those with higher PG fit have higher professional efficacy.*”

We hope that we have thoroughly addressed the concerns that the reviewer has, and look forward to your decision.

Yours sincerely,

Guohong Li

School of Public Health, Shanghai Jiao Tong University School of Medicine, Shanghai, China

Center for HTA, China Hospital Development Institute, Shanghai Jiao Tong University, Shanghai, China

guohongli@sjtu.edu.cn
